# Treatment and outcome in metastatic lobular breast cancer in the prospective German research platform *OPAL*

**DOI:** 10.1007/s10549-023-06882-7

**Published:** 2023-02-18

**Authors:** M. Thill, M.-O. Zahn, A. Welt, E. Stickeler, A. Nusch, T. Fietz, J. Rauh, N. Wetzel, L. Kruggel, M. Jänicke, N. Marschner, N. Harbeck, A. Wöckel, T. Decker, the OPAL study group

**Affiliations:** 1 Agaplesion Markus Krankenhaus, Frankfurt, Germany; 2 MVZ Onkologische Kooperation Harz, Goslar, Germany; 3 Universitätsklinikum Essen, Essen, Germany; 4 Universitätsklinikum Aachen, Aachen, Germany; 5 Praxis für Hämatologie und internistische Onkologie, Ratingen, Germany; 6 Schwerpunktpraxis für Hämatologie und internistische Onkologie, Singen, Germany; 7 Gemeinschaftspraxis Innere Medizin, Witten, Germany; 8 iOMEDICO, Freiburg, Germany; 9 Praxis für interdisziplinäre Onkologie und Hämatologie, Freiburg, Germany; 10 Brustzentrum, Frauenklinik LMU Klinikum, Munich, Germany; 11 Universitätsklinikum Würzburg, Würzburg, Germany; 12 Onkologie Ravensburg, Praxis für Hämatologie und Onkologie GbR, Ravensburg, Germany

**Keywords:** Breast neoplasms, Registries, Treatment outcome, Prospective studies

## Abstract

**Purpose:**

Evidence about routine treatment and outcome of patients with invasive lobular cancer (ILC) is limited, especially regarding metastatic disease. Here we present prospective real-world data of patients with metastatic ILC (mILC) as compared to patients with metastatic invasive ductal cancer (mIDC) receiving systemic therapy in routine care in Germany.

**Methods:**

Prospective data on patient and tumor characteristics, treatments, and outcomes of patients with mILC (*n* = 466) and mIDC (*n* = 2100), recruited between 2007 and 2021 into the Tumor Registry Breast Cancer/OPAL were analyzed.

**Results:**

Compared to mIDCs, patients with mILC were older at start of first-line treatment (median 69 vs. 63 years) and had more often lower grade (G1/G2: 72.8% vs. 51.2%), hormone receptor (HR)-positive (83.7% vs. 73.2%) and less often HER2-positive (14.2% vs. 28.6%) tumors, which metastasized more frequently to the bone (19.7% vs. 14.5%) or peritoneum (9.9% vs. 2.0%), and less frequently to the lungs (0.9% vs. 4.0%). Median OS of patients with mILC (*n* = 209) and mIDC (*n* = 1158) was 30.2 months [95% confidence interval (CI) 25.3, 36.0] and 33.7 months [95% CI 30.3, 37.9], respectively. Multivariate survival analysis did not show a significant prognostic impact of the histological subtype [HR mILC vs. mIDC 1.18 (95% CI 0.97–1.42)].

**Conclusion:**

Overall, our real-world data confirm clinicopathological differences between mILC and mIDC breast cancer patients. Despite patients with mILC presenting with some favorable prognostic factors, ILC histopathology was not associated with a better clinical outcome in multivariate analysis, suggesting the need for more tailored treatment strategies for patients with the lobular subtype.

## Introduction

With a relative frequency of about 10–15%, invasive lobular cancer (ILC) is the second most common histological subtype of invasive breast cancer [1–3]. ILC is known to differ from the more frequent invasive ductal cancer (IDC) in molecular, clinical and pathological characteristics [4–6] and increasing evidence suggests that ILC should be regarded as a distinct disease requiring further research to optimize therapeutic approaches [4, 7]. However, current guidelines and clinical trials do not distinguish between IDC and ILC in treatment pathways.

Many studies show that ILC is associated with lower histological grade, hormone receptor (HR)-positivity, human epidermal growth factor receptor 2 (HER2−) negativity [4–6], higher proportions of multifocal, multicentric and bilateral spread [8, 9] and a different metastatic pattern [10] as compared to IDC. Whether differences between the histological subtypes have an impact on their clinical outcomes remains unclear. Pathologic response rates after neoadjuvant chemotherapy have been reported to be lower among patients with ILC but might not be well correlated with their further outcome [11, 12]. Results on overall survival of patients with ILC have been controversial, showing either no prognostic impact of the histological subtype [8, 13], a better prognosis for patients with ILC [12, 14–16] or a worse prognosis for ILC as compared to IDC [17–24]. Besides these conflicting results from studies mainly focusing on early breast cancer, evidence from studies considering patients with metastatic breast cancer (mBC) is very limited.

To add knowledge on ILC-subtype mBC and its treatment in routine care, and to evaluate whether ILC histology is an independent prognostic factor in mBC, we analyzed demographic, treatment, and outcome data of patients with mBC documented in a large German breast cancer registry.

## Methods

OPAL is an open, non-interventional, prospective, multi-center tumor registry focusing on changes in treatment reality of patients with early and advanced (locally advanced and/or metastatic) breast cancer. It started in 2017 and continues the Tumor Registry Breast Cancer (TMK, NCT01351584) [25], which has recruited patients with breast cancer since 2007. OPAL was approved by the responsible ethics committee and is registered at ClinicalTrials.gov (NCT03417115). For this analysis, only patients with mBC, recruited at start of their first palliative systemic treatment, were considered to avoid overestimation of outcome data. Eligible patients were enrolled at 221 study sites in Germany, including breast centers, comprehensive cancer centers, hospitals or office-based oncological and gynecological outpatient practices. Detailed information on patient and tumor characteristics, physician-reported factors influencing treatment decisions, biomarker testing and all neoadjuvant, adjuvant and current systemic treatments as well as radiotherapies and surgeries were collected, and patients were followed until death or up to 5 years from enrolment. Further methods of the registry have been previously described by Fietz et al. [25].

For this study we compared treatment, as well as demographic, clinical, and outcome data of OPAL/TMK registry patients with mBC in the subgroups with invasive ductal cancer (corresponding to invasive cancer of *no special type* (NST) but named according to the common term used at the time of recruitment) or invasive lobular breast cancer who received first-line treatment. Descriptive statistics and survival methods (Kaplan–Meier, Cox regression analysis) were used to compare groups and outcomes (SAS for Windows, Version 9.4. SAS Institute Inc., Cary, NC, USA). Start point to calculate survival was the beginning of first-line systemic treatment for mBC. For the assessment of demographic, disease-related and treatment data, mBC-patients with start of treatment until 2021 were included. To obtain sufficient long-term outcome data, survival analysis was performed in patients with start of treatment until June 2016, with potential follow-up until 2021.

A Cox proportional hazards model was used to investigate potential independent prognostic factors for survival. The following independent variables, prospectively selected based on literature and clinical relevance for either treatment decision making (potential confounding by indication) or baseline factors prognostic for overall survival, were incorporated into the model: age at start of first-line, ECOG performance status, Charlson Comorbidity Index, hormone receptor status at inclusion, HER2 status at inclusion, grading at primary diagnosis, tumor stage at primary diagnosis, metastasis at start of first-line and histology. No variable selection was performed. Confidence intervals (CI) for the regression coefficients were based on the Wald statistics. All presented *P* values are two-sided, 5% will be interpreted as significant. There were no multiplicity adjustments to the level of significance.

## Results

### Cohort definition

**Fig. 1:**
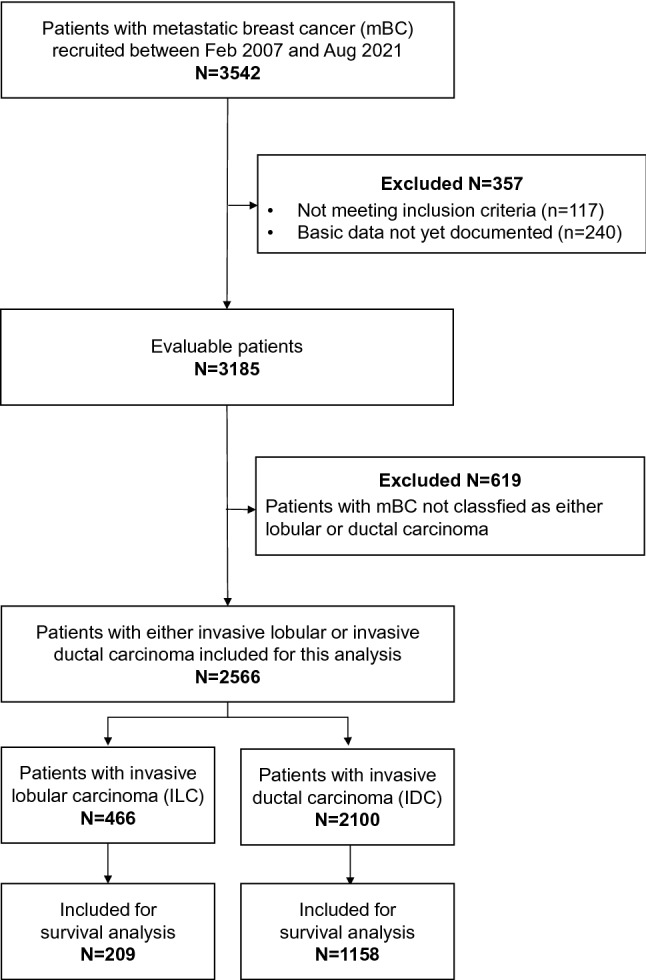
Cohort definition

Data cut-off for the present analysis was August 31, 2021. By then, 3542 patients with mBC had been recruited, of whom 3185 were evaluable for analysis (Fig. 1). For the present analysis, only patients with either lobular (*N* = 466) or ductal (*N* = 2100) histology were included and considered for assessment of demographic, disease-related and treatment data (*n* = 2566). For analysis of survival data, 1367 patients (*N* = 209 mILC and *N* = 1158 mIDC), who had been recruited until June 2016 and followed up until August 31, 2021, were included, to obtain sufficient long-term outcome data.

### Patient and tumor characteristics

Table 1 presents patient and tumor characteristics of 2566 patients included into this analysis according to documented histological type. At start of their first-line treatment, patients with mILC were (in median) 6 years older than patients with mIDC (69 vs. 63 years). Comorbidity as documented by the Charlson Comorbidity Index (CCI) was comparable between mILC and mIDC patients. At the time of study inclusion, lobular tumors were more often HR− positive (estrogen and/or progesterone receptor (ER/PR) positive: 83.7% vs. 73.3%) and less often HER2-positive (14.2% vs. 28.6%) as compared to mIDCs. Histological grade was more frequently G1/2 in the mILC group (72.8% vs. 51.2%), while the occurrence of primary metastatic disease (tumor stage IV at diagnosis) was similar in both groups (mILC: 38.2%, mIDC: 36.6%). The type of metastasis at the start of first-line treatment was more often bone-only (19.7% vs. 14.5%) or peritoneum (9.9% vs. 2.0%) and overall, less often visceral (58.2% vs. 65.8%) in the mILC group.

**Table 1:** Patient and tumor characteristics of 2566 patients with metastatic invasive lobular or metastatic invasive ductal cancer

Characteristic	Invasive lobular (*N* = 466)		Invasive ductal (*N* = 2100)	
	Median	25–75% Quartile	Median	25–75% Quartile
Median age at start of first-line (years)	68.6	58.7–75.7	62.9	53.1–72.5

	*N*	%	*N*	%
ECOG performance status at inclusion				
ECOG 0	134	28.7	666	31.7
ECOG ≥ 1	176	37.8	693	33.0
Unknown/missing^a^	156	33.5	741	35.3
Comorbidity at inclusion (CCI)^b^				
CCI 0	370	79.4	1753	83.5
CCI ≥ 1	96	20.6	347	16.5
Unknown/missing	–	–	–	–
ER/PR status at inclusion				
HR-positive	390	83.7	1537	73.2
ER positive PR positive	290	62.2	1164	55.4
ER positive, PR negative	98	21.0	328	15.6
ER negative, PR positive	2	0.4	45	2.1
ER negative, PR negative	62	13.3	507	24.1
Unknown/missing	14	3.0	56	2.6
HER2 status at inclusion				
Positive	66	14.2	601	28.6
Negative	399	85.6	1494	71.1
Unknown/missing	1	0.2	5	0.2
Grading at diagnosis				
G1	26	5.6	87	4.1
G2	313	67.2	988	47.1
G3/4	96	20.6	884	42.1
GX/unknown/missing	31	6.6	141	6.7
Tumor stage at diagnosis of BC				
0	4	0.9	17	0.8
I	29	6.2	227	10.8
II	115	24.7	534	25.4
III	96	20.6	351	16.7
IV	178	38.2	769	36.6
Unknown	44	9.4	202	9.6
Metastasis at start of first-line therapy^c^				
Any metastasis	466	100	2100	100
Bone only	92	19.7	305	14.5
Liver only	20	4.3	125	6.0
Lung only	4	0.9	84	4.0
Peritoneum (leading metastatic site)	46	9.9	41	2.0
Non-visceral metastases—other^d^	38	8.2	149	7.1
Visceral metastases—other	201	43.1	1130	53.8
Unknown	65	13.9	266	12.7

*BC* breast cancer; *ECOG* Eastern Cooperative Oncology Group; *ER* estrogen receptor; *HER2* human epidermal growth factor receptor 2; *HR* hormone receptor; *PR* progesterone receptor

^a^Collection of data on ECOG performance status started in 2011 and data are missing for patients recruited prior to 2011

^b^Charlson Comorbidity Index (CCI) according to Quan [28, 29]

^c^Metastasis at start of palliative 1st-line therapy (8 weeks before to 4 weeks after start of 1st-line treatment)

^d^Non-visceral: skin, bone and/or lymph node metastasis except bone-only

### Treatment

The first-line treatments of patients with mBC according to the histological type and receptor status are shown in Table 2.

**Table 2:** First-line therapy in 2566 patients with metastasized breast cancer according to the histological subtype (mILC and mIDC)

	Invasive lobular (*N* = 466)		Invasive ductal (*N* = 2100*)	
	*N*	%	*N*	%
HR-positive/HER2-negative patients				
2007–2021	348		1176	
ET alone	106	30.4	321	27.3
ET + CDK4/6-inhibitor	159	45.7	395	33.6
CTx (including targeted therapy)	83	23.9	460	39.1
2007–2016	146		641	
ET	79	54.1	278	43.4
CTx (including targeted therapy)	67	45.9	363	56.6
2017-2021^a^	202		535	
ET alone	27	13.4	49	9.2
ET + CDK4/6-inhibitor	159	78.7	389	72.7
CTx (including targeted therapy)	16	7.9	97	18.1
HER2-positive patients				
2007–2021	66		601	
Double anti-HER2-blockade	8	12.1	85	14.1
Single anti-HER2-blockade	47	71.2	444	73.9
No anti-HER2 blockade	11	16.7	72	12.0
2007–2012	26		203	
Anti-HER2-blockade	20	76.9	175	86.2
No anti-HER2-blockade	6	23.1	28	13.8
2013-2021^b^	40		398	
Double anti-HER2-blockade	8	20.0	82	20.6
Single anti-HER2-blockade	27	67.5	272	68.3
No anti-HER2 blockade	5	12.5	44	11.1
TNBC patients				
2007–2021	52		322	
Platinum-based therapy	12	23.1	54	16.8
PD-L1-inhibitor	4	7.7	27	8.4
Other	36	69.2	241	74.8
2007–2019	32		240	
Platinum-based therapy	7	21.9	43	17.9
Other	25	78.1	197	82.1
2019-2021^c^	20		82	
PD-L1-inhibitor	4	20.0	27	32.9
Other	16	80.0	55	67.1

*CTx* chemotherapy; *ET* endocrine therapy; *HER2* human epidermal growth factor receptor 2; *HR* hormone receptor; *PD-L1* programmed cell death-ligand 1

*For one patient with ductal carcinoma, receptor status could not be assigned

^a^After approval of CDK4/6-inhibitors

^b^After approval of pertuzumab

^c^After approval of the PD-L1 inhibitor atezolizumab

In the HR-positive-/HER2-negative subgroup, treatment was assessed separately before and after 2016 (approval of CDK4/6-i). The proportion of endocrine therapies (ET) was slightly higher in the mILC group throughout the study period from 2007 to 2021, whereas patients with mIDC were more often treated with chemotherapy (CTx). After the implementation of CDK4/6-i, the majority of mILC and mIDC patients (78.7%, *n* = 159 and 72.7%, *n* = 389) received ET together with CDK4/6-i, while the proportion of patients receiving ET only and CTx clearly declined in both subgroups: ET only was reduced from 54.1% (*n* = 79) to 13.4% (*n* = 27) among mILCs and from 43.4% (*n* = 278) to 9.2% (*n* = 49) among mIDCs. CTx declined from 45.9% (*n* = 67) to 7.9% (*n* = 16) within the mILC subgroup and from 56.6% (*n* = 363) to 18.1% (*n* = 97) among the mIDC subgroup.

The proportion of HER2-positive patients who did not receive anti-HER2-targeted therapies throughout the study period was higher in mILCs as compared to mIDCs (*n* = 11, 16.7% vs. *n* = 72, 12.0%), whereas the difference between the groups was markedly reduced over time.

Slightly fewer triple negative breast cancer (TNBC) patients with mILC received anti-PD-L1 guided therapy after 2019 as compared to TNBC patients with mIDC (*n* = 4, 20.0% vs. *n* = 27, 32.9%). Of note, sample size was very small both among TNBC and HER2-positive patients with mILC, which must be considered when interpreting the data.

### Survival

**Fig. 2:**
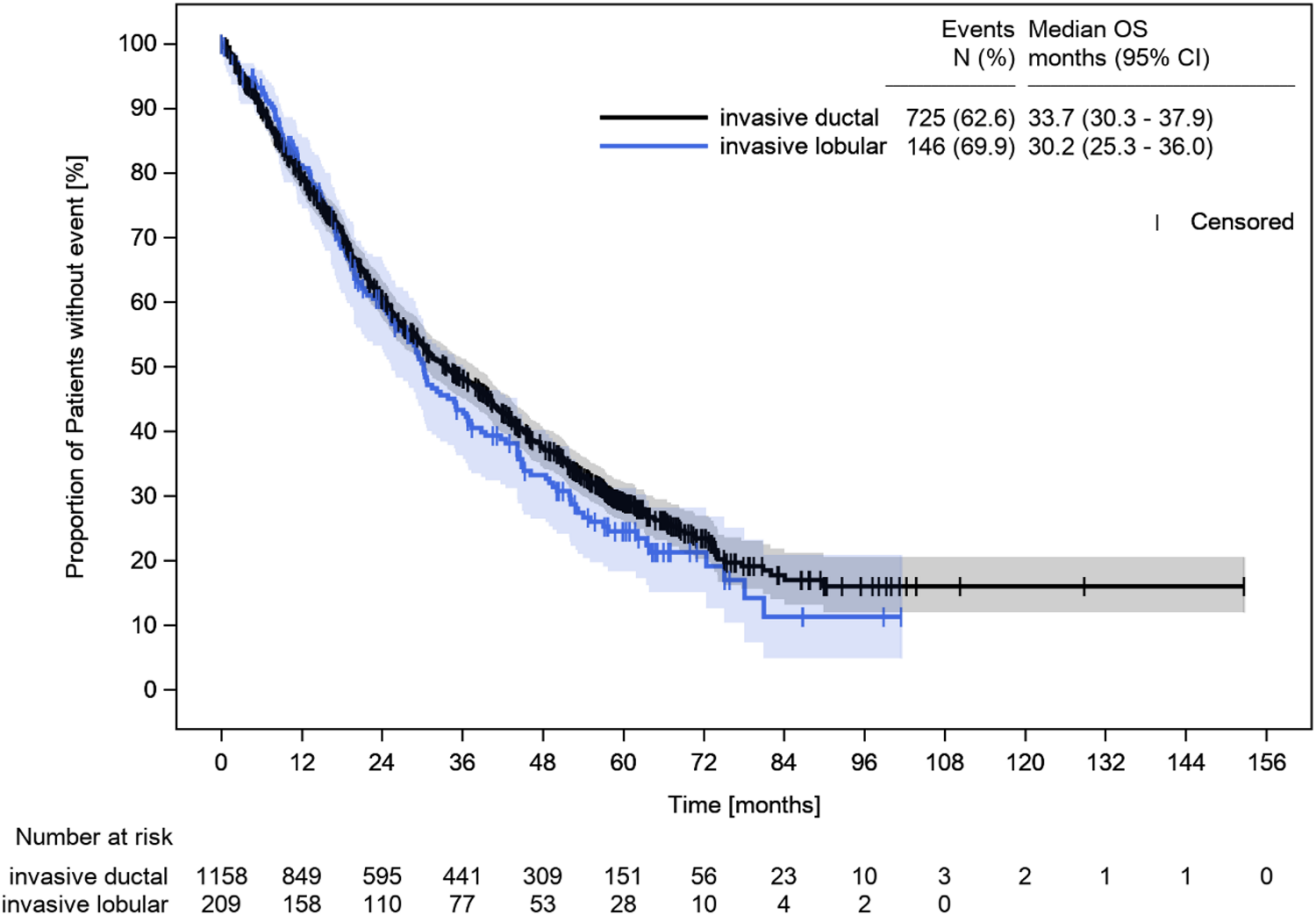
Overall survival of patients with metastatic invasive lobular vs. invasive ductal breast cancer. All patients starting first-line treatment until 2016 (follow-up of up to 4 years until 2020) were included. *CI* confidence interval; *OS* overall survival

As depicted in Fig. 2, median OS of patients with mILC (*n* = 209) across all receptor subtypes was 30.2 months [(95% Confidence Interval (CI) 25.3–36.0 months), 69.9% events] and 33.7 months [(95% CI 30.3–37.9 months), 62.6% events] for mIDC patients (*n* = 1158).

**Fig. 3:**
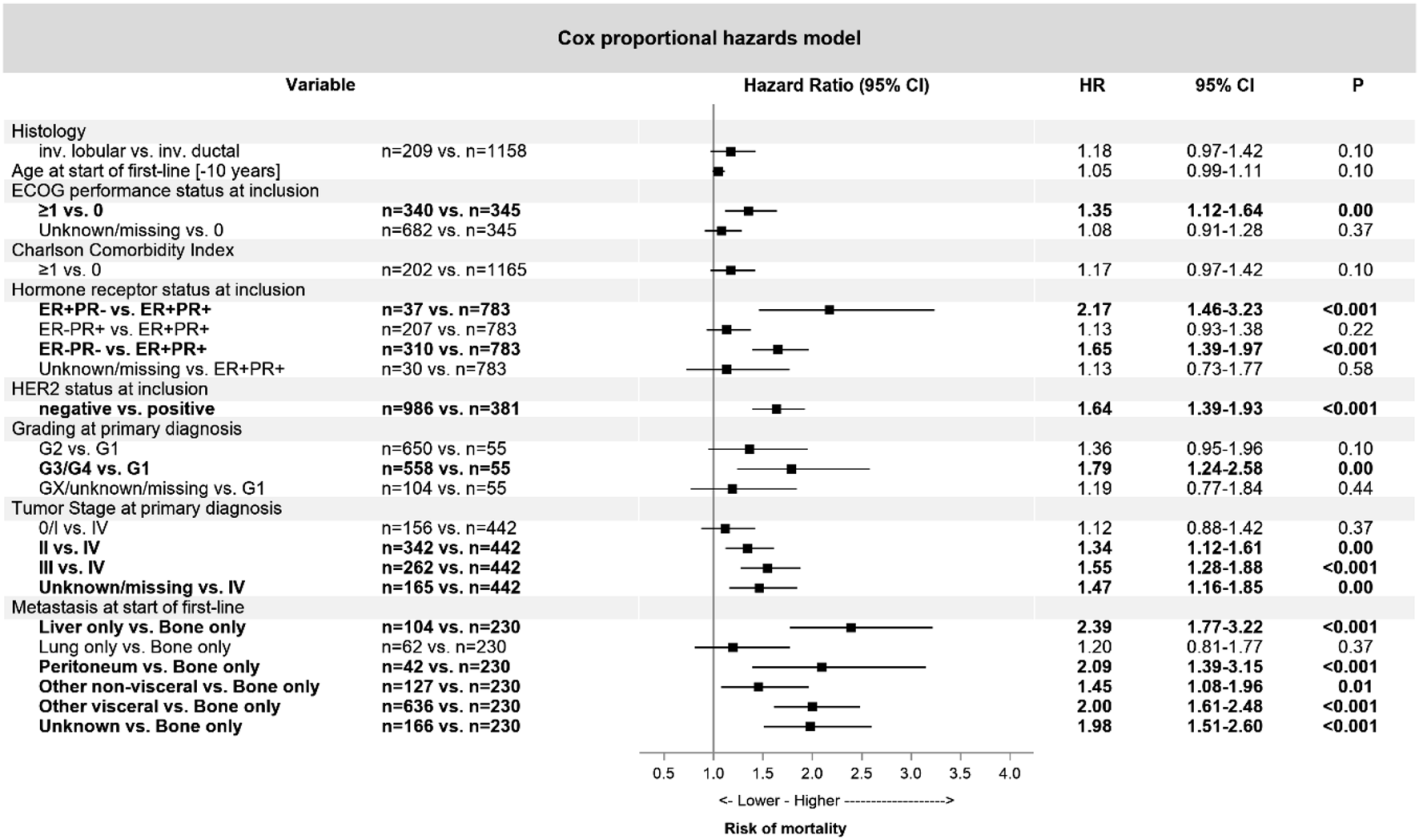
Multivariate survival analysis (Cox regression) in 1367 patients with metastatic breast cancer. *CI* confidence interval; *ER* estrogen receptor; *HR* hazard ratio; *HER2* human epidermal growth factor receptor 2; *PR* progesterone receptor

Multivariate survival analysis of the entire group (*n* = 1367; Fig. 3) did not show a significant impact of the histological subtype on survival [Hazard Ratio/HR 1.18 (95% CI 0.97–1.42); *p* = 0.1]. Higher ECOG performance status at start of therapy, negative HR-status, lack of HER2 overexpression, high tumor grade, high tumor stage at initial diagnosis, and metastatic sites other than bone-only (liver, peritoneum, other visceral or non-visceral) were all independently associated with a worse prognosis.

## Discussion

To our knowledge, we present the largest multi-center study using prospectively documented data of over 2500 patients with de novo or recurrent mILC and mIDC. The lobular subtype is found in approximately 10–15% of patients with invasive breast cancer [1] and therefore represents a relevant subgroup in view of the high overall incidence of BC. Despite known specific characteristics of lobular carcinomas [4, 6], current guidelines do not provide specific treatment recommendations for this subtype and evidence regarding routine treatment and outcome, especially for mBC, is very limited.

To add insights to possible differences between IDC and ILC in the metastatic setting, we analyzed characteristics, treatments and outcome in 2566 patients (1367 with regard to OS) with mBC treated since 2007 in routine care (> 80% by office-based oncologists and gynecologists) and documented in a large German tumor registry [25]. Patients with mILC represented about 15% of all evaluable patients. Consistent with previous findings from retrospective studies, patients with mILC presented at an older age [13, 18, 22–24, 26], and their tumors were more likely to be of lower grade [13, 18, 19, 23, 26], HR-positive [19, 22, 23, 26] and less likely to be HER2-positive [13, 19, 22, 23, 26] compared to patients with mIDC. We also observed, as have others, that lobular tumors were found to metastasize more frequently to the bone or peritoneum and less frequently to the lung compared to ductal tumors [13, 19, 22, 24]. So far, reasons for this different metastatic pattern are largely unclear. It has been hypothesized that the loss of cell-adhesion molecule E-cadherin, which is typical for ILC [4], may have an influence on the metastatic spread of tumor cells in lobular tumors [8], and that differences in metastatic behavior of the histologic subtypes may affect treatment-decision making in real-life [13]. Besides differences in the metastatic pattern, the unique molecular portrait of lobular tumors has generally been suggested to be responsible for the poor chemo-sensitivity of ILC and good response to ET [5, 27]. Against this background, treatment decisions in ILC have often been made in favor of ET [7]. While patients with HR-positive/HER2-negative tumors with mILC histology were still treated less often with CTx and more often with ET only as compared to mIDC patients with HR-positive/HER2-negative tumors, the majority of patients in both subgroups were treated with ET in combination with CDK4/6-i after their approval, demonstrating the rapid implementation of newly approved therapies and guidelines into routine care.

Previously reported results concerning outcome of ILC compared to IDC have been contradictory, and differences regarding study design and study population, such as the inclusion of early breast cancer patients [8, 12, 14–20, 22–24] or the combined analysis of both, pure and mixed lobular carcinomas [13] hinder comparison between published studies and the present results.

Recently, two retrospective studies evaluated the impact of the histological subtype on real-world outcomes of patients with advanced breast cancer [21, 26].

Watanabe et al. [21] found ILC histology to be associated with poorer prognosis as compared to IDC in multivariate analysis among patients with de novo stage IV breast cancer, however the study was only performed at a single institution and limited in sample size, including 29 patients with ILC. Further, the study was restricted to patients with ER positive and HER-2 negative disease [21]. Using data from 18 academic cancer centers, more than 13,000 patients, thereof 1804 with mILC, could be included into a French multicentre cohort study [26]. Consistent with Watanabe et al. [21], patients with mILC had a worse prognosis as compared to patients with mIDC in multivariable adjusted analysis [26], which is in contrast to our results, as no significant influence of the histopathological subtype could be demonstrated in our multivariable adjusted analysis. While the authors of the French study could include prognostic tumor characteristics and age in their multivariate analysis, no information on performance status or comorbidities was available. This could explain the different results compared to our study, as it cannot be ruled out that patients with ILC in the French cohort were in a worse overall condition than patients with IDC. Owing to the study inclusion period, few patients of the French cohort received CDK4/6-i, which is nowadays the preferred treatment option in patients with HR-positive/HER2-negative disease, representing the majority of mILC patients, as it was also reflected in our data. The authors state that therefore no conclusion can be drawn on the influence of CDK4/6-i treatment on patients outcome [26]. This is also a limitation of our data, as only patients recruited until 2016, i.e., before the implementation of CDK4/6-i, could be included in survival analyses with sufficient long-term outcome data. Furthermore, only patients receiving antineoplastic therapy were included into the study [25]. While the interpretation of data from cohort studies is limited by the lack of randomization, population-based studies, as the present, provide important and valuable evidence on routine practice patterns and the implementation of guidelines into routine care. Further strengths of our study are the prospective documentation, the relatively large patient number, a clear definition of included disease stage (mBC only at start of first-line therapy) and the comprehensive adjustment for possible prognostic factors, including hormone receptor status.

## Conclusion

In summary, our prospectively documented real-word data confirm previously reported clinicopathological differences between patients with mILC and mIDC. Nevertheless, and despite patients with ILC presenting with some favorable prognostic features, the results of multivariable adjusted Cox regression analyses did not show a significant impact of ILC histology on prognosis of patients with mBC. Whether the development towards treatment with CDK4/6-i in patients with HR-positive/HER2-negative disease (who represent the majority of mILC patients), may have an influence on their overall survival remains to be clarified. As such, there is a high demand for population-based studies investigating the impact of histological subtypes, while also considering patients with metastatic disease. Research within this field remains crucial for an improved understanding of the lobular breast cancer subtype to enable the development of more tailored treatment strategies.

## Data Availability

The datasets generated during and analysed during the current study are not publicly available because of data privacy restrictions.

## Abbreviations

BC: Breast cancer
CCI: Charlson Comorbidity Index
CDK4/6-i: Cycline-dependent kinase 4/6-inhibitors
CI: Confidence interval
CTx: Chemotherapy
ECOG: Eastern Cooperative Oncology Group
ET: Endocrine therapy
HER2: Human epidermal growth factor receptor 2
HR: Hormone receptor
IDC: Invasive ductal cancer
ILC: Invasive lobular cancer
m: Metastatic
mBC: Metastatic breast cancer
NST: No special type
OS: Overall survival
PD-L1: Programmed cell death-ligand 1
TMK: Tumor Registry Breast Cancer
TNBC: Triple negative breast cancer
